# Retrospective multicenter study of elderly patients with platinum-sensitive relapsed ovarian cancer treated with trabectedin and pegylated liposomal doxorubicin (pld) in a real-world setting: a geico study

**DOI:** 10.1186/s12885-024-12577-z

**Published:** 2024-07-05

**Authors:** María Jesús Rubio, Aránzazu Manzano, Luis Miguel de Sande, Purificación Estévez-García, María del Mar Gordon, Diego Soto de Prado, Blanca Hernando Fernández de Aranguiz, Eva M. Guerra-Alia, Anna Carbó-Bagué, Ignacio Romero, Miguel Corbellas, Alba González-Haba, Carlos E. Robles-Barraza, Jerónimo Martínez-García, Antonio González-Martín

**Affiliations:** 1 Oncology Department, Hospital Universitario Reina Sofía, Av. Menéndez Pidal, Córdoba, 14004 Spain; 2 Oncology Department, Hospital Clínico San Carlos, Madrid, Spain; 3 Oncology Department, Hospital Universitario de León, León, Spain; 4 Oncology Department, Hospital Universitario Virgen del Rocío, Sevilla, Spain; 5 Oncology Department, Medical Oncology Department, Hospital Universitario de Jerez de la Frontera, Hospital Virgen Macarena, Jerez de la Frontera, Sevilla, Spain; 6 Oncology Department, Hospital Clínico Universitario de Valladolid, Valladolid, Spain; 7 Oncology Department, Hospital Universitario de Burgos, Burgos, Spain; 8 Oncology Department, Hospital Universitario Ramón y Cajal, Madrid, Spain; 9 Institut Català d’Oncologia (ICO), Girona, Spain; 10 Oncology Department, Fundación Instituto Valenciano de Oncología, Valencia, Spain; 11 Oncology Department, Hospital Universitario Dr. Peset, Valencia, Spain; 12 Oncology Department, Hospital Universitario de Badajoz, Badajoz, Spain; 13 Unidad de Gestion Clinica Oncología Integral, Hospital Universitario Nuestra Señora de Valme, Sevilla, Spain; 14 Oncology Department, Hospital Clínico Universitario V. de la Arrixaca, Murcia, Spain; 15 Oncology Department, Clínica Universidad de Navarra, Pamplona, Spain

**Keywords:** Ovarian cancer, Elderly, Trabectedin, Real-world studies

## Abstract

**Background:**

Trabectedin in combination with pegylated liposomal doxorubicin (PLD) is approved for the treatment of patients with platinum-sensitive relapsed ovarian cancer. Nevertheless, there is currently limited information regarding this treatment in elderly patients with ovarian cancer in a real-world setting.

**Methods:**

This observational and multicentric study retrospectively evaluated trabectedin plus PLD in a real-world setting treatment of elderly patients diagnosed with platinum-sensitive relapsed ovarian cancer, treated according to the Summary of Product Characteristics (SmPC) from 15 GEICO-associated hospitals. Patients ≥ 70 years old at the time of treatment initiation and platinum-free intervals ≥ 6 months were considered eligible.

**Results:**

Forty-three patients with a median age of 74.0 years were treated between January 1st, 2015, and December 31st, 2019 in 15 Spanish centers. Four patients achieved complete response (9.3%), 14 (32.6%) partial response, and 13 (30.2%) stable disease as the best radiological response. In the analysis of biological overall response according to CA125 serum levels (i.e., Rustin criteria), 14 responded to the treatment (32.6%), 11 responded and normalized (25.6%), three patients stabilized (7.0%) and three progressed (7.0%). Median progression-free survival (PFS) and overall survival (OS) in the study population were 7.7 and 19.5 months, respectively. The most common grade 3/4 adverse events were neutropenia (*n* = 8, 18.7%) and asthenia (*n* = 5, 11.6%).

**Conclusions:**

This analysis demonstrated that trabectedin combined with PLD is a feasible and effective treatment in elderly patients with platinum-sensitive relapsed ovarian cancer, showing an acceptable safety profile, which is crucial in the palliative treatment of these patients.

**Supplementary Information:**

The online version contains supplementary material available at 10.1186/s12885-024-12577-z.

## Background

Ovarian cancer is the third most common gynecological cancer with 313,959 new diagnoses and 207,252 annual deaths worldwide [1, 2]. The data show that approximately 50% of ovarian cancer is diagnosed in women over 65 years and is expected that in a growing elderly population, this percentage will increase in the next decades as life expectancy improves [3]. The prognosis of ovarian cancer is usually poor, with a 5-year survival rate of 43% and just 25% in women aged over 75 years [4]. In this population, comorbid conditions and frailty could be related to elevated mortality incidence that may impede optimal management [5]. In fact, age is a known prognostic factor in ovarian cancer, but the optimal treatment of elderly patients with ovarian cancer has not been determined yet and represents an utmost challenge [6]. However, the current treatment landscape for advanced epithelial ovarian cancer has seen significant advancements with the integration of Poly (ADP-ribose) polymerase (PARP) inhibitors. These drugs have proven particularly effective for patients with defects in DNA repair pathways, such as BRCA mutations, although the challenge of resistance to PARP inhibitors remains, prompting ongoing research into new combinations and strategies to enhance their efficacy, even in platinum-resistant cases [7].

These patients have often a complex medical history which limits their probability of undergoing surgery. Chemotherapy is frequently associated with higher toxicity rates and, although different regimens have been proposed, literature shows somewhat contradictory results [8–10]. Additionally, although 80% of newly diagnosed ovarian cancer patients respond to first-line platinum-based chemotherapy, its effectiveness and clinical benefit are reduced in each subsequent line due to the development of platinum resistance and cumulative toxicity [11]. Furthermore, standard doses and schedules of chemotherapy are not appropriate for many elderly women [8], and patients over 75 years are inadequately represented in clinical trials [6].

In this context, trabectedin (Yondelis^®^) is an antitumor agent that binds to the minor groove of DNA, providing an anti-inflammatory and anti-angiogenic activity to modulate the tumor microenvironment [12]. It is a synthetic drug originally isolated from the sea squirt *Ecteinascidia turbinata*. Trabectedin in combination with pegylated liposomal doxorubicin (PLD) is approved in the European Union [13] and in ~ 70 other countries around the globe for the treatment of patients with platinum-sensitive recurrent ovarian cancer (ROC). The approval was based on the results of the multicenter, randomized phase III OVA-301 study, in which the combination of trabectedin plus PLD vs. PLD alone showed statistically significant and clinical benefits in patients with ROC in progression-free survival (PFS) [14, 15].

These beneficial results have been further proved in additional clinical trials [16], and also under routinary clinical practice [17–19]. However, the median age of the included patients does not exceed 62 years in clinical trials, 59.8 years in OVC-3006 study [16], or 66 years in real-world settings [61.3 years in NIMES-ROC study [16], 64 years in PROSPECTYON study [18] and 66 years in OVA-YOND study [19]].

There are no specific studies for elderly patients, aged ≥ 70 years, and their representation in the literature is limited. In addition, most data regarding the profile of elderly patients with ROC and their outcomes following the treatment with the combination are reported as results of subgroup analyses. Therefore, the objective of the current study was to describe the real-life clinical practice in elderly patients with ROC treated with trabectedin combined with PLD.

## Methods

### Study design

This retrospective, observational, multicentric study included elderly patients diagnosed with platinum-sensitive relapsed ovarian cancer who received at least one cycle of trabectedin (Yondelis^®^) treatment in combination with PLD, following the Summary of Product Characteristics (SmPC) and local clinical practice in Spain.

According to the terms of the marketing authorization, trabectedin was administered every three weeks at a recommended dose of 1.1 mg/m² as an intravenous infusion over 3 h immediately after 30 mg/m² of PLD. Eligible participants included women aged ≥ 70 years with platinum-sensitive ROC, defined as disease relapse after ≥ 6 months after completion of last platinum-containing therapy, who have received a minimum of one cycle of trabectedin and PLD before their inclusion in the study, and who signed an informed consent document. Excluded were patients who had no available their medical records and patients who explicitly refused to participate in the study.

### Ethics

All women who could be interviewed in the hospital (i.e., accessible, alive patients) signed the written informed consent to participate in the study. Informed consent was not required from inaccessible patients (those who died before the analysis or who were not possible to contact) according to ethics committee permissions and applicable law for retrospective studies in Spain. All study procedures were conducted in accordance with the ethical standards as laid down in the 1964 Declaration of Helsinki and its later amendments and were approved by the Ethics Committees of Hospital Reina Sofía-Provincial de Córdoba (reference number: 5111) according to national legislation. Data was carried out according to the Spanish Order SAS/3470/2009, from December 16th, which publishes the guidelines for observational studies, and the Law 14/2007, of July 3rd, on Biomedical Research. Data were recorded following Good Clinical Practice compliance at each hospital, respecting the Regulation (EU) 2016/679 of the European Parliament and the Council of April 27th of 2016 on data protection.

### Outcome measures

For this analysis, we collected clinical characteristics of the patients and medical history including sex, age, relevant comorbidities, previous history of non-ovarian cancer, number of previous relapses, family history of cancer, performance status as per Eastern Cooperative Oncology Group (ECOG) score, blood tests, platinum sensitivity, and platinum-free interval (PFI). Data related to trabectedin and PLD treatment, such as duration of treatment, number of cycles, doses, and reasons to end the treatment were also registered.

The primary endpoint was to describe the real-life use of trabectedin plus PLD in elderly patients diagnosed with platinum-sensitive ROC treated according to the SmPC. Secondary endpoints included the overall response rate (ORR), overall survival (OS), disease control rate (DCR), PFS, trabectedin combined with PLD treatment information, prior and subsequent treatments, patient characteristics/medical history and safety profile. The ORR was defined as the percentage of patients with a complete response (CR) or partial response (PR) according to the Response Evaluation Criteria in Solid Tumors (RECIST) v.1.1 [20]. The DCR was calculated as the percentage of patients achieving an ORR and/or stable disease (SD). The OS was defined as the time interval from the first administration of trabectedin combined with PLD until death due to any cause. PFS was determined as a time in months from the first administration of trabectedin with PLD until radiological progression (or death due to any cause) according to RECIST v.1.1 [20].

Information about the treatment with trabectedin combined with PLD and previous and subsequent therapies were also registered and analyzed. The biological overall response was defined according to CA125 serum levels (Rustin/GCIG criteria) [21].

The safety profile of trabectedin combined with PDL treatment was assessed as all treatment-related hematological and non-hematological, serious, and non-serious adverse events (AEs). All AEs were graded according to Common Terminology Criteria for Adverse Events (CTCAE) 4.03.9.

### Data analysis

The sample size was determined by all patients diagnosed with platinum-sensitive ROC treated with trabectedin and PLD during the study period. As a multicenter study at a national level and considering that a percentage of failures in the collection or analysis of the samples (missing or unevaluable data) of ~ 10.0%, a sample size of 40–45 patients was estimated.

## Results

### Patient disposition and characteristics

From January 1st, 2015 to December 31st, 2019, a total of 46 patients who received at least one cycle of trabectedin plus PLD were enrolled by 15 GEICO (Grupo Español de Investigación en Cáncer Ginecológico)-associated hospitals with expertise in gynecological cancers across Spain. Three patients were excluded from the analysis set because of screening failures (i.e., PFI < 6 months in two patients and age < 70 years in another), thus, overall, 43 patients were analyzed in the study (Table 1). The median age of patients at diagnosis was 71.0 (range: 62–85 years), whereas the median age at the treatment start was 74.0 (range: 70–86 years), with 69.8% of patients aged between 70 and 75 years and 30.2% of patients older than 75 years. At the time of diagnosis, most patients had high-grade serous ovarian cancer (*n* = 40, 93.0%), mostly presenting FIGO (International Federation of Gynecology and Obstetrics system) stage IIIC (*n* = 22, 51.2%) (Table 1). The median PFI of patients was 9 months (range: 6–42). The 81.4% (*n* = 35) and 18.6% (*n* = 8) of patients had a PFI of 6–12 months and > 12 months, respectively. BRCA mutation was found in seven patient (16.3%). Due to the retrospective nature of the study, it was unknown if the number of patients with this genetic recombination might be higher. Most patients presented comorbidities (*n* = 33, 76.7%), among which the most common were arterial hypertension (*n* = 18, 41.9%), dyslipidemia (*n* = 13, 30.2%) and depression (*n* = 8, 18.6%) (Table 1). Medical history of six patients (14.0%) also included prior breast cancer (*n* = 3, 7%), carcinoid tumor in the appendix (*n* = 1, 2.3%), peritoneal carcinomatosis (*n* = 1, 2.3%) and thyroid cancer (*n* = 1, 2.3%). Overall, 30 patients (69.8%) had a family history of tumors with breast cancer (*n* = 14, 32.5%) as the most common familiar one followed by colon (*n* = 7, 16.2%), gastric and pancreatic cancer (*n* = 6, 13.9% each). At study entry, most patients presented an ECOG performance status score of 0–1 (72.1%, *n* = 31). Before trabectedin plus PLD treatment, most patients had measurable (*n* = 35, 81.4%) or bulky disease (*n* = 23, 53.5%). No brain metastasis were found in the patients included in this study.

**Table 1:** Patient and disease characteristic

	Patients (*N* = 43)
Age at trabectedin + PLD treatment initiation, median (range)	74.0 (70–86)
Relevant comorbidities, n (%)	33 (76.7)
Arterial hypertension	18 (41.9)
Dyslipidemia	13 (30.2)
Depression	8 (18.6)
Diabetes mellitus	4 (9.3)
Hypothyroidism	3 (7)
Anxiety	1 (2.3)
Arthrosis	1 (2.3)
COPD	1 (2.3)
Cerebrovascular disease	1 (2.3)
Obesity	1 (2.3)
Other relevant	33 (76.7)
Previous cancer type, n (%)	6 (14.0)
Breast	3 (7)
Carcinoid tumor in appendix	1 (2.3)
Peritoneal carcinomatosis	1 (2.3)
Thyroidal cancer	1 (2.3)
Family history of cancers, n (%)	30 (69.8)
Number of previous relapses, median (range)	2.0 (1–5)
ECOG PS, n (%)*	
0	14 (32.6)
1	17 (39.5)
2	4 (9.3)
Unknown	6 (14.0)
Hematological data, n (%)	
Platelet count (cells/nL), median (range)**	241,000 (12^4^-42^4^)
Leukocytes (10^9^/L), median (range)**	6.5 (4–14)
Absolute neutrophils (cells/nL), median (range)**	3820 (1830–8880)
Hemoglobin (g/dL), median (range)^#^	12.1 (8–15)
CA 125 (U/mL), median (range)**	174.9 (0-4389)
Platinum sensitivity, n (%)	43 (100.0)
Platinum-Free Interval, median (range)	9.0 (6–42)
Platinum-Free Interval, n (%)	
0–6 months	0 (0.0)
6–12 months	35 (81.4)
More than 12 months	8 (18.6)
Age at ovarian cancer diagnostic, median (range)	71.0 (62–85)
Tumor histology, n (%)***	
High-grade serous ovarian cancer	40 (93.0)
High-grade fallopian tube cancer	1 (2.3)
High-grade primary peritoneal cancer	1 (2.3)
FIGO stage, n (%)****	
IA	2 (4.7)
IC1	1 (2.3)
IIIA1	2 (4.7)
IIIA2	1 (2.3)
IIIB	3 (7.0)
IIIC	22 (51.2)
IVB	5 (11.6)
Unknown	4 (9.3)
BRCA mutation, n (%)*****	7 (16.3)

CA 125: cancer antigen 125; COPD: Chronic Obstructive Lung Disease; ECOG: Eastern Cooperative Oncology Group; ECOG PS: ECOG performance status; FIGO: International Federation of Gynecology and Obstetrics; PLD: pegylated liposomal doxorubicin.

* Data of 2 patients (4.7%) are missing

**Data of 6 patients (14.0%) are missing

# Dara of 4 patients (9.3%) are missing

***Data of 1 patient (2.3%) is missing

****Data of 3 patients (7.0%) is missing

*****Unknown if tested due to the retrospective study

### Previous surgery and systemic treatments

Overall, 38 patients had undergone one (*n* = 30, 69.8%) surgery, with 24 patients undergoing primary debulking surgery (55.8%), 15 patients interval debulking surgery (34.8%) and six patients secondary cytoreduction (13.9%; Supplementary Table 1). Surgery outcomes corresponded to resection R0 in 22 (47.8%) or R1 and R2 in 11 patients (23.9%). Patients were pretreated with a median of two prior chemotherapy lines (range: 1–5), 39.5% of whom were pretreated with one or two lines of chemotherapy prior to trabectedin plus PLD. Furthermore, the 16.3%, 2.3% and 2.3% of patients were treated with three, four and five previous lines of chemotherapy, respectively (Supplementary Table 1). Most patients were treated with either carboplatin-paclitaxel combination (*n* = 36, 83.7%) or bevacizumab (*n* = 16, 37.2%) and the combination of carboplatin-PLD (*n* = 14, 32.6%).

### Extent of exposure

Patients received a median of five cycles (range: 1–21 cycles) over a median treatment duration of 4.0 months (range: 0–21), with 20 (46.5%) patients receiving six or more cycles of trabectedin plus PLD (Table 2). Trabectedin was administered in a median total dose of 1.8 mg/m^2^ (range: 1–28). Most patients (*n* = 35, 81.4%) received trabectedin at an initial dose of 1.1 mg/m^2^, although 16.3% started treatment at a reduced dose (< 1.1 mg/m^2^). The median total dose of PLD was 50.0 mg/m^2^ (range: 34–865). Most patients (*n* = 34, 79.1%) received an initial dose of PLD of 30.0 mg/m^2^ yet, eight patients (18.3%) started treatment at a reduced dose (< 30 mg/m^2^).

**Table 2:** Trabectedin and PLD treatment

	Patients (*N* = 43)
Duration of treatment in months, median (range)	4.0 (0–21)
Number of cycles, median (range)	5.0 (1–21)
Number of cycles, n (%)	
1	2 (4.7)
2	8 (18.6)
3	6 (14.0)
4	3 (7.0)
5	4 (9.3)
6	11 (25.6)
7	1 (2.3)
8	2 (4.7)
9	1 (2.3)
>9	5 (11.6)
Trabectedin	
Initial dose trabectedin (mg/m^2^), median (range)	1.1 (0.8–1.5)
Total dose trabectedin (mg/m^2^), median (range)**	1.8 (1–28)
Initial dosing split, n (%)	
<1.1	7 (16.3)
1.1	35 (81.4)
>1.1	1 (2.3)
PLD	
Initial dose of PLD (mg/m^2^), median (range)	30.0 (23–46)
Total dose PLD (mg/m^2^), median (range)**	50.0 (34–865)
Initial dosing split	
<30	8 (18.6)
30	34 (79.1)
>30	1 (2.3)
Reason to end of treatment, n (%)	
Progression	17 (39.5)
Doctor’s decision	10 (23.3)
Toxicity	10 (23.3)
Patient’s decision	3 (7.0)
Others	3 (7.0)

PLD: pegylated liposomal doxorubicin

* Data of 1 patient (2.3%) is missing

** Data of 6 patients (14.0%) is missing

One patient (2.3%) had a dose interruption due to concomitant medication received at start of treatment during trabectedin and PLD treatment, whereas 19 patients (44.2%) had no treatment delay, the rest experienced a median delay of 6.5 days (range: 0–54 days). The most common cause for the end of treatment was progression (*n* = 17, 39.5%), followed by the doctor’s decision and toxicity (*n* = 10, 23.3% each), patient’s decision or other reasons (*n* = 3, 7.0% each).

### Effectiveness of trabectedin treatment

**Fig. 1:**
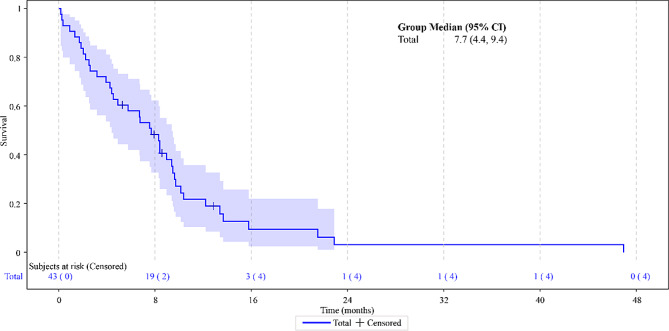
Progression-free survival

**Fig. 2:**
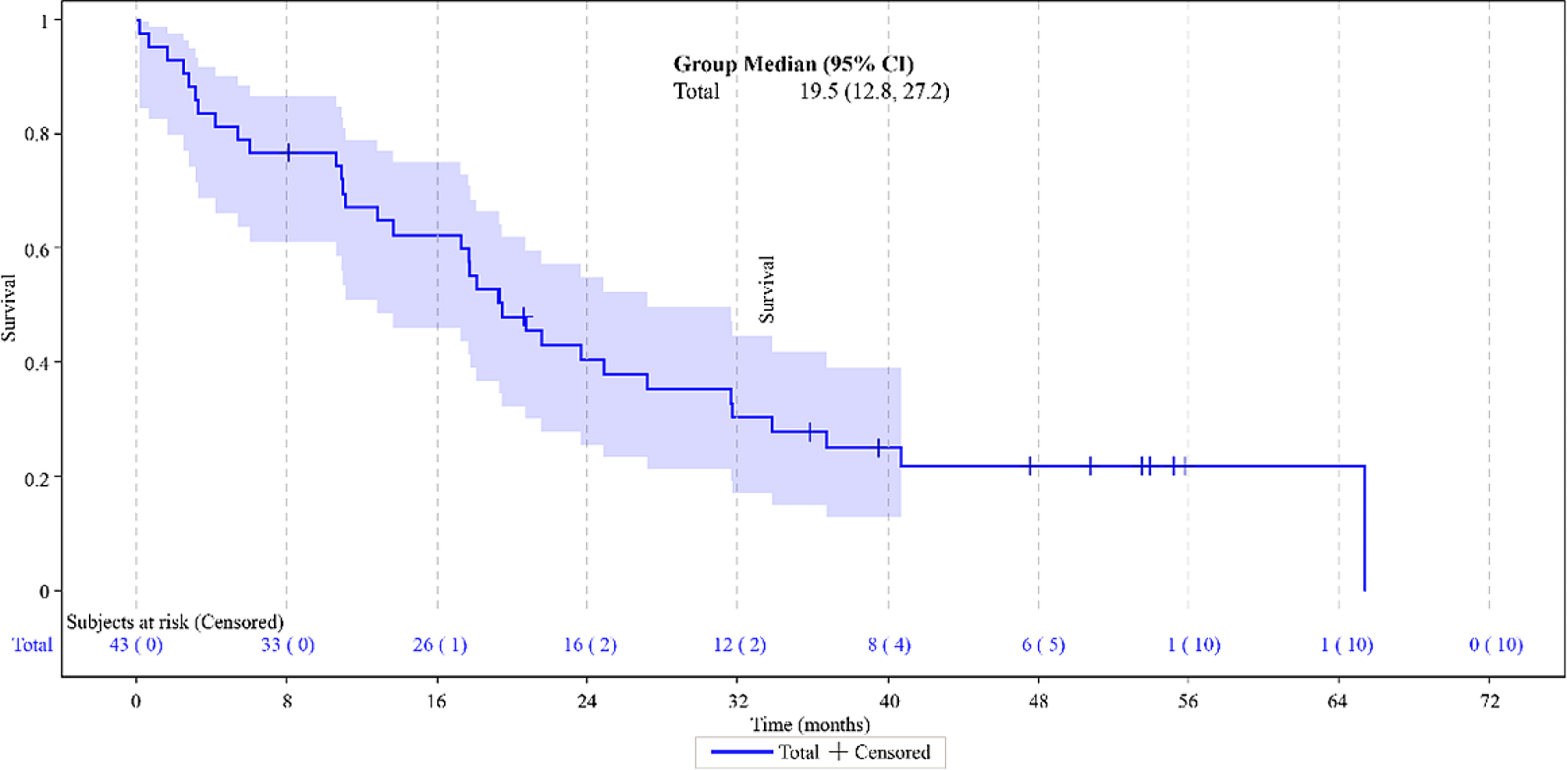
Overall survival

The median PFS in the overall study population was 7.7 months (95% confidence interval [CI]: 4.4–9.4) (Fig. 1) and the median OS was 19.5 months (95% CI: 12.8–27.2) (Fig. 2). As per the radiological analysis of response, four patients achieved a CR (9.3%) and 14 (32.6%) patients obtained a PR, reaching an ORR of 41.9%. Additionally, 13 patients (30.2%) had disease stabilization as the best response for a DCR of 72.1% (Table 3).

In an analysis of the biological best response, 14 patients responded to the treatment (32.6%), 11 responded and achieved normalization (25.6%), three patients progressed (7.0%) and three disease stabilization (7.0%; Table 3).

**Table 3:** Best response to trabectedin plus PLD treatment

	Patients (*N* = 43)
Radiological best overall response, n (%)	
Complete response	4 (9.3)
Partial response	14 (32.6)
Stable Disease	13 (30.2)
Progression Disease	5 (11.6)
Not assessable	7 (16.3)
Biological best overall response, n (%)	
Response	14 (32.6)
Response and normalization	11 (25.6)
Stabilization	3 (7.0)
Progression	3 (7.0)
Not assessable	12 (27.9)
Objective response rate (ORR), n (%)	
PR or CR	18 (41.9)
NE	7 (16.3)
Disease control rate (DCR), n (%)	
PR or CR or SD	31 (72.1)
NE	7 (16.3)

CI: confidence interval; CR: complete response; DCR: disease control rate; NE: not estimated; ORR: overall response rate; PD: progression disease; PR: partial disease; SD: stable disease

### Safety profile

Asthenia, neutropenia, nausea, and anemia were the most commonly observed AEs. The most frequently reported grade 1/2 AEs included asthenia (*n* = 16, 37.2%), neutropenia (*n* = 7, 16.3%) anemia (*n* = 9, 20.9%) nausea (*n* = 8, 18.6%), and vomiting (*n* = 5, 11.6%) (Table 4). Twenty-five grade 3/4 AEs were reported, with the most common being neutropenia (*n* = 8, 18.7%) and asthenia (*n* = 5, 11.6%) (Table 4). No deaths attributed to treatment-related AEs or unexpected AEs occurred.

**Table 4:** Adverse events, categorized by the highest grade per patient within each grade group, reported during treatment with trabectedin in combination with PLD.

Hematology term	Total	I-II	III	IV
Hematological, n (%)				
Neutrophil count decreased	15 (34.9)	7 (16.3)	2 (4.7)	6 (14.0)
Anemia	10 (23.3)	9 (20.9)	1 (2.3)	0 (0.0)
Platelet count decreased	5 (11.6)	2 (4.7)	3 (7.0)	0 (0.0)
White blood cells decreased	1 (2.3)	1 (2.3)	0 (0.0)	0 (0.0)
Non-hematological				
Asthenia	21 (48.8)	16 (37.2)	5 (11.6)	0 (0.0)
Nausea	9 (20.9)	8 (18.6)	1 (2.3)	0 (0.0)
Mucositis	6 (14.0)	4 (9.3)	2 (4.7)	0 (0.0)
Vomiting	6 (14.0)	5 (11.6)	1 (2.3)	0 (0.0)
Transaminitis	4 (9.3)	2 (4.7)	2 (4.7)	0 (0.0)
Urinary tract infection	3 (7.0)	3 (7.0)	0 (0.0)	0 (0.0)
Constipation	2 (4.7)	2 (4.7)	0 (0.0)	0 (0.0)
Hypertension	2 (4.7)	2 (4.7)	0 (0.0)	0 (0.0)
Skin Disorders	2 (4.7)	2 (4.7)	0 (0.0)	0 (0.0)
Abdominal pain	1 (2.3)	1(2.3)	0 (0.0)	0 (0.0)
Alopecia	1 (2.3)	1(2.3)	0 (0.0)	0 (0.0)
Dissociated Cholestasis	1 (2.3)	1(2.3)	0 (0.0)	0 (0.0)
Dysuria	1 (2.3)	1(2.3)	0 (0.0)	0 (0.0)
General muscular pain	1 (2.3)	1(2.3)	0 (0.0)	0 (0.0)
Headache	1 (2.3)	1(2.3)	0 (0.0)	0 (0.0)
Hiporexia	1 (2.3)	1(2.3)	0 (0.0)	0 (0.0)
Hypokalemia	1(2.3)	1(2.3)	0 (0.0)	0 (0.0)
Palmar-plantar erythrodysesthesia	1 (2.3)	1 (2.3)	0 (0.0)	0 (0.0)
Respiratory infection	1 (2.3)	0 (0.0)	1 (2.3)	0 (0.0)
Serum Urea Increased	1 (2.3)	1 (2.3)	0 (0.0)	0 (0.0)

### Subsequent therapies

After the study end, 32 (74.4%) patients received a subsequent antineoplastic treatment (74 treatment options) (Supplementary Table 2). Some patients were treated with more than one line. The median number of the posterior treatment lines was 2 (range: 1–5). Regarding all the administered treatments, the most common ones were the combination of carboplatin plus paclitaxel (*n* = 12, 15.6%), followed by carboplatin-gemcitabine (*n* = 9, 11.7%) and olaparib (*n* = 7, 9.1%). The 63,7% of the patients were treated witth others antineoplastic treatments. Overall, the best response to these treatments was CR in one patient (1.4%), PR in 21 (28.4%), and SD in 27 (36.5%). 18 patients progressed (18.9%) and 11 patients were not evaluated (14.9%).

## Discussion

Ageing process is different for each individual, hence, the chronological threshold at which an adult is considered “old” is not well defined [22]. In oncology, the age of 70 is broadly considered as a chronological landmark and it is frequently used for the definition of elderly patients. The risk of ovarian cancer increases with age, especially after 50 years of age [23]. Moreover, despite the improvement in survival, patients > 60 years are considered to have a poor prognosis [24]. In this subgroup toxicity risk of chemotherapy is higher due to the number of comorbidities that elderly patients typically have. In addition, elderly patients with ovarian cancer are widely undertreated [4] and underrepresented in clinical trials [25] considering that only 10% of participants aged ≥ 70 years are represented in phase III trials [26]. Consequently, it is difficult to establish evidence-based clinical recommendations for this patients’ subset [22]. For instance, a study from the FRANCOGYN group of the National College of French Gynecologists and Obstetricians (CNGOF) showed that women with similar tumor characteristics have different treatments depending on age [4]. Elderly women aged ≥ 75 years with ovarian cancer are particularly sub-optimally treated in both, surgery and chemotherapy as they underwent fewer intestinal resections compared to those between 70 and 74 years [27]. Similarly, in contrast to younger patients, elderly women are not treated with standard front-line chemotherapy (carboplatin/paclitaxel) [27], in spite of the fact that it is reported that the treatment with this combination increase survival in comparison with single-agent carboplatin also in the elderly population (210). In general, the relapse rate after the end of the standard chemotherapy in women with ovarian cancer is high, and reach around 23% during the first 6 months, while 60% relapse after this period [28, 29]. For patients with platinum-sensitive ROC, the combined treatment of trabectedin and PLD have demonstrated its antitumor activity, efficacy, and safety in different studies [14–19]. Yet, data from the elderly population are still limited since no other study has been exclusively performed in these patients and the presence of this subgroup is generally scarce in previous studies.

The present study provides further evidence of the effectiveness of the combination in elderly patients diagnosed with platinum-sensitive ROC in a real-world setting. Noteworthy, the patients’ median age at the start of the treatment was 74.0 years with a range of 70–86 years and most patients received trabectedin plus PLD as second or third-line chemotherapy (79%). In this population, trabectedin combined with PLD treatment showed a median PFS and OS of 7.7 months (95% CI: 4.4–9.4) and 19.5 months (95% CI: 12.8–27.2), respectively. In addition, 72.1% of the patients achieved a disease control rate. Other studies such as OVA-301 [13, 14] and OVC-3006 [16] studies, different retrospective analyses [30, 31], case series [32–35] and real-world evidence, including ProspectYon [18], OVA-YOND [19], and NIMES-ROC [17], have previously demonstrated that trabectedin plus PLD is an effective treatment for platinum-sensitive ROC in a wider population. In OVA-301, the efficacy and safety of trabectedin plus PLD was compared with that of PLD alone in 672 women with ROC after failure of first-line, platinum-based chemotherapy. For platinum-sensitive patients, median PFS was 9.2 months vs. 7.5 months, respectively (HR, 0.73; 95% CI, 0.56 to 0.95; *p* = 0.0170). In that study, 80 patients aged ≥ 65 years and treated with trabectedin plus PLD improved PFS over 106 patients treated with PLD alone (6.3 vs. 5.8 months, hazard ratio (HR): 0.92, 95% CI: 0.62–1.35) in a similar way that patients aged < 65 years (7.4 vs. 5.9, HR: 0.75, 95% CI: 0.59–0.95) [14]. Concerning the survival results from OVA-301, subgroup analyses based on baseline variables, performed to evaluate their impact on the OS results, all showed the trend in an OS treatment effect in favor of trabectedin plus PLD except of impact of age ≥ 65 years as compared with < 65 years [15]. In general population of patients from OVA-301 the most common grade 3/4 AEs reported in patients treated with trabectedin plus PLD were transient neutropenia, ALT increase, leukopenia and thrombocytopenia. A safety analysis of data from OVA-301 reported that the safety profile of trabectedin plus PLD was virtually identical between the subjects younger or older than 65 years, except for more fatigue in the older subset compared with younger patients (< 65 years: 14% vs. ≥ 65 years: 7%) [36, 37].

Pignata et al. have recently reported the results of the observational, prospective, phase IV NIMES-ROC study of trabectedin plus PLD in real-life clinical practice [17]. Among 218 enrolled patients, 32.1% were ≥ 65–74 years old and 8.7% were aged ≥ 75 years. The study population represented a very heterogeneous and heavily pretreated population who received up to eight prior chemotherapy lines (72.5% of patients were pretreated with ≥ 2 prior chemotherapy lines) prior to trabectedin plus PLD. After receiving a median of 6 cycles of trabectedin plus PLD (range: 1–24), in the global population, the median PFS was 9.5 months (CI 95%: 7.9–10.9) and the OS was 23.6 months (95% CI: 18.1–34.1). Although, the results were not specified according to the age of the patients, the impact of different prognostic variables was reported. In this regard, the impact of age on PFS had a HR of 0.849 (95% CI: 0.618–1.166) in patients < 65 compared to ≥ 65 years (*p* = 0.3112). Similarly, the age also had no significant impact (HR = 0.668; 95% CI: 0.419–1.065, *p* = 0.0902) on median OS when comparing both subgroups.

In this real-world study, despite the fact that elderly patients were suffering from numerous comorbidities and that they were heavily pretreated, the results were comparable with previous literature, demonstrating again a relevant efficacy in these fragile patients. It is also necessary to mention that the population in real-world trials is less restricted than in clinical studies, being more representative of the effectiveness in clinical practice.

In terms of safety, in the present study, trabectedin plus PLD was shown to be safe and well-tolerated. The most frequently reported AEs were mild to moderate and included asthenia (48.8%), neutropenia (34.9%) and anemia (23.3%). Only six patients presented grade 4 AEs (14.0%), and all of them were neutropenia. This safety profile for trabectedin plus PLD treatment was consistent with prior studies, with no new safety signals reported (36–38).

The primary weakness of this analysis pertains to its retrospective nature, which makes it challenging to establish cause-and-effect relationships between various variables and the sample size of the study. In addition, the sample size is small due to the difficulty in finding more patients who meet the inclusion criteria. Moreover, due to the non-interventional setting of this study, certain data are unknown or missing and this may further hamper the interpretation of the results.

The findings from the study hold significant implications for both clinical practice and future research in the treatment of ovarian cancer. In terms of clinical practice, the study underscores that trabectedin combined with PLD is an effective and well-tolerated treatment option specifically for elderly patients dealing with platinum-sensitive recurrent ovarian cancer. This combination therapy offers clinicians a viable alternative to consider when devising treatment plans for this particular patient group. Importantly, the study highlights that trabectedin plus PLD can be administered safely even in elderly patients with underlying health conditions, providing oncologists with crucial guidance in decision-making for this demographic. Moreover, the study contributes valuable real-world evidence supporting the use of trabectedin plus PLD in clinical settings. By focusing on elderly patients with recurrent ovarian cancer, a population often underrepresented in research, the study addresses a gap in the literature and enhances our understanding of treatment outcomes in this context. In terms of its contribution to existing literature, the study enriches our knowledge by presenting data on the efficacy and safety of trabectedin plus PLD specifically in elderly patients with recurrent ovarian cancer. This demographic, characterized by advanced age and multiple prior treatment regimens, presents unique challenges and considerations that the study addresses comprehensively. Looking ahead to future research, there are several avenues for exploration. Longitudinal studies could provide insights into the long-term outcomes and quality of life implications associated with trabectedin plus PLD treatment in elderly patients. Comparative studies against other standard treatments or combinations could further optimize treatment strategies tailored to this specific patient population. Additionally, research into predictive biomarkers or genetic factors influencing response to trabectedin plus PLD could pave the way for more personalized approaches in ovarian cancer treatment, advancing precision medicine in the field.

## Conclusions

This real-world data study provides valuable information about the effect of trabectedin plus PLD treatment in elderly patients (≥ 70 years) with platinum-sensitive ROC. The results consistently support the efficacy of the treatment regardless the advanced age and administration of previous lines of treatment. In addition, this combination showed a manageable safety profile despite the high presence of comorbidities among elderly patients. The overall data observed are consistent with those obtained in the general population demonstrating its potential as an alternative to treat this frail subgroup.

### Electronic supplementary material

Below is the link to the electronic supplementary material.

Supplementary Material 1

## Data Availability

Data supporting the findings of this study are available from the corresponding author upon reasonable request.

## Abbreviations

AE: Adverse event
CNGOF: National College of French Gynecologists and Obstetricians
CTCAE: Common Terminology Criteria for Adverse Events
CR: Complete response
DCR: Disease control rate
ECOG: Eastern Cooperative Oncology Group
FIGO: International Federation of Gynecology and Obstetrics system
GEICO: Grupo Español de Investigación en Cáncer Ginecológico
ORR: Overall response rate
OS: Overall survival
PR: Partial response
PLD: Pegylated liposomal doxorubicin
PFI: Platinum free interval
PFS: Progression free survival
ROC: Recurrent ovarian cancer
RECIST: Response Evaluation Criteria in Solid Tumors
SD: Stable disease
SmPC: Summary of Product Characteristics
