# Supplementary material for: Retrospective multicenter study of elderly patients with platinum-sensitive relapsed ovarian cancer treated with trabectedin and pegylated liposomal doxorubicin (pld) in a real-world setting: a geico study
Source: BMC Cancer. 2024 Jul 5;24:803. doi: 10.1186/s12885-024-12577-z (PMC11225319; doi:10.1186/s12885-024-12577-z)
Supplement: Supplementary file 1 — Supplementary Material 1 [file 12885_2024_12577_MOESM1_ESM.docx]

**Supplementary Table 1**. Previous surgery and systemic treatments against ovarian cancer

|  | **Patients**  (N=43) |
| --- | --- |
| Surgeries, median (range)* | 1.0 (1-2) |
| Surgeries performed before relapse, n (%) | 40 (87.0) |
| Surgery type, n (%)** |  |
| Primary debulking surgery | 24 (55.8) |
| Interval debulking surgery | 15 (34.8) |
| Secondary debulking surgery | 6 (13.9) |
| Surgery outcome, n (%)*** |  |
| R0 | 22 (47.8) |
| R>0 | 11 (23.9) |
| Unknown | 13 (28.3) |
| Number of previous treatments lines per patient, median (range) | 2.0 (1-5) |
| 1 prior line | 17 (39.5) |
| 2 prior lines | 17 (39.5) |
| 3 prior lines | 7 (16.3) |
| 4 prior lines | 1 (2.3) |
| 5 prior lines | 1 (2.3) |
| Most common previous systemic treatments, n (%) |  |
| Bevacizumab | 16 (37.2) |
| Carboplatin-Caelyx | 14 (32.6) |
| Carboplatin-Paclitaxel | 36 (83.7) |
|  | |

*Data of 5 patients (11.6%) is missing

** Data of 2 patients (4.3%) is missing

*** Data of 1 patient (2.1%) is missing

**Supplementary Table 2**. Subsequent treatments against ovarian cancer. Values were calculated over all responses on lines, not over the number of patients.

|  | **Patients**  (N=43) |
| --- | --- |
| Number of posterior treatments lines* | 32 |
| Most common subsequent treatments, n (%) |  |
| Carboplatin-Paclitaxel | 12 (15.6) |
| Carboplatin-gemcitabine | 9 (11.7) |
| Olaparib | 7 (9.1) |
| Posterior treatments lines, n (%) |  |
| 0 | 11 (25.6) |
| 1 | 9 (20.9) |
| 2 | 8 (18.6) |
| 3 | 10 (23.3) |
| 4 | 3 (7.0) |
| 5 or more lines | 2 (4.7) |
| Best Response, n (%) |  |
| Complete response | 1 (1.4) |
| Partial response | 21 (28.4) |
| Stable Disease | 27 (36.5) |
| Progression | 14 (18.9) |
| Not assessable | 11 (14.9) |
| Reason to end treatment, n (%) |  |
| Progression | 33 (44.6) |
| Toxicity | 15 (20.3) |
| Physician’s decision | 17 (23.0) |
| Completed | 2 (2.7) |
| Patient’s decision | 1 (1.4) |
| Death | 1 (1.4) |
| Other | 4 (5.4) |
| Unknown | 1 (1.4) |
|  | |

SD: standard deviation

*Data of 11 patients (25.6%) is missing
